# Using a Rapid Learning Health System for Stratified Care in Emerging Adult Mental Health Services: Protocol for the Implementation of Patient-Reported Outcome Measures

**DOI:** 10.2196/51667

**Published:** 2024-03-20

**Authors:** Gina Dimitropoulos, David Lindenbach, Melissa Potestio, Tom Mogan, Amanda Richardson, Alida Anderson, Madison Heintz, Karen Moskovic, Jason Gondziola, Jessica Bradley, Haley M LaMonica, Frank Iorfino, Ian Hickie, Scott B Patten, Paul D Arnold

**Affiliations:** 1 Mathison Centre for Mental Health & Education University of Calgary Calgary, AB Canada; 2 Faculty of Social Work University of Calgary Calgary, AB Canada; 3 Alberta Health Services Edmonton, AB Canada; 4 Brain and Mind Centre The University of Sydney Sydney Australia; 5 Department of Community Health Sciences University of Calgary Calgary, AB Canada

**Keywords:** learning health system, stratified care, patient-reported outcome measures, mental health, emerging adults, protocol papers, pragmatic clinical trials, e-mental health, RE-AIM, Reach, Effectiveness, Adoption, Implementation, and Maintenance, implementation science, adult, health system, stratified care, treatment, implementation, acceptability, measurement-based care

## Abstract

**Background:**

Mental illness among emerging adults is often difficult to ameliorate due to fluctuating symptoms and heterogeneity. Recently, innovative approaches have been developed to improve mental health care for emerging adults, including (1) implementing patient-reported outcome measures (PROMs) to assess illness severity and inform stratified care to assign emerging adults to a treatment modality commensurate with their level of impairment and (2) implementing a rapid learning health system in which data are continuously collected and analyzed to generate new insights, which are then translated to clinical practice, including collaboration among clients, health care providers, and researchers to co-design and coevaluate assessment and treatment strategies.

**Objective:**

The aim of the study is to determine the feasibility and acceptability of implementing a rapid learning health system to enable a measurement-based, stratified care treatment strategy for emerging adults.

**Methods:**

This study takes place at a specialty clinic serving emerging adults (age 16-24 years) in Calgary, Canada, and involves extensive collaboration among researchers, providers, and youth. The study design includes six phases: (1) developing a transdiagnostic platform for PROMs, (2) designing an initial stratified care model, (3) combining the implementation of PROMs with stratified care, (4) evaluating outcomes and disseminating results, (5) modification of stratified care based on data derived from PROMs, and (6) spread and scale to new sites. Qualitative and quantitative feedback will be collected from health care providers and youth throughout the implementation process. These data will be analyzed at regular intervals and used to modify the way future services are delivered. The RE-AIM (Reach, Effectiveness, Adoption, Implementation, and Maintenance) framework is used to organize and evaluate implementation according to 3 key objectives: improving treatment selection, reducing average wait time and treatment duration, and increasing the value of services.

**Results:**

This project was funded through a program grant running from 2021 to 2026. Ethics approval for this study was received in February 2023. Presently, we have developed a system of PROMs and organized clinical services into strata of care. We will soon begin using PROMs to assign clients to a stratum of care and using feedback from youth and clinicians to understand how to improve experiences and outcomes.

**Conclusions:**

This study has key implications for researchers and clinicians looking to understand how to customize emerging adult mental health services to improve the quality of care and satisfaction with care. This study has significant implications for mental health care systems as part of a movement toward value-based health care.

**International Registered Report Identifier (IRRID):**

PRR1-10.2196/51667

## Introduction

### Background

Most mental disorders emerge before the age of 25 years, resulting in a considerable burden of disease across the life span [1,2]. Emerging adulthood (age 16-24 years) represents a unique stage in life [3], characterized by low mental health service use and high treatment attrition rates [4,5]. Emerging adults are also burdened by perceptions of stigma and embarrassment surrounding help-seeking behaviors, which is further worsened by the lack of mental health literacy in this population [6,7].

### Transdiagnostic Stratification of Mental Illness

Symptoms of mental illness in emerging adults are often evolving and do not always fit neatly into diagnostic categories created for older adults [8]. Rapid changes in symptoms make it difficult to conduct accurate diagnostic assessments and select appropriate treatments, especially when there are significant delays between first contact with a health care provider and the beginning of treatment [9].

Adopting a transdiagnostic approach to mental illness in young adults may improve assessment and treatment [10-12]. Transdiagnostic assessments focus on identifying the severity of mental illness and symptom domains, which cut across conventional diagnoses rather than diagnosing a discrete disorder [8,11]. Likewise, transdiagnostic treatments use shared principles of evidence-based therapies to help clients manage a range of symptoms [13].

Another emerging method to improve youth mental health treatment is through an approach whereby standardized patient-reported outcome measures (PROMs) of mental health are used to monitor treatment progress. A growing body of literature is showing how adopting PROMs in mental health care settings improves clinical outcomes, improves communication between patients and providers, and reduces treatment attrition, although the evidence supporting integrating PROMs into clinical decision-making is stronger among older adults relative to young people [14-18]. To date, mental health providers have been slow to embrace the routine use of PROMs even though many acknowledge the potential benefits [19].

Pairing the implementation of PROMs with a transdiagnostic approach facilitates the use of “stratified care.” Stratified and stepped care represent 2 different models of care with differing approaches to selecting the most appropriate interventions for patients. With “stepped care,” all patients begin with lower-intensity treatments and change to higher-intensity treatments if they do not respond [20]. In contrast, stratified care patients with more severe illness (and associated functional impairment) will receive appropriately more intensive treatment from the outset [20,21]. Recent randomized clinical trials of depression and anxiety indicate that the stratified care approach resulted in better clinical outcomes than stepped care or standard care [22,23].

### Rapid Learning Health Systems

The implementation of a stratified care model in clinical settings is complicated by the fact that it is difficult for individual clinics to understand how to assign clients to strata and how to select which services should be available in each stratum. One potential way to implement stratified care in a routine clinical setting is through the adoption of a rapid learning health system (RLHS). The RLHS was first proposed by Etheredge [24] and involves the use of health records to determine the best treatment options in a personalized care environment based on the specific needs of each presenting patient. By comparing a patient’s health records to individuals in similar medical situations, health practitioners unlock new capabilities to compare treatment effectiveness, adopt best practices, assess results, and provide feedback from their unique case [25]. Furthermore, as evidence is formulated through information supplied by typical health care patients rather than clinical trial participants selected based on highly specific criteria, the RLHS may not face issues of generalizability that are typically associated with randomized clinical trials [24]. As such, this lends credence to the use of the RLHS in biomedical practice, where clients are heterogenous, time and resource constraints are common, and treatment planning needs to evolve dynamically to fit the situation.

An RLHS has been shown to improve the quality, efficiency, and cost-effectiveness of health care delivery across a range of patients, medical conditions, and settings [26]. For example, in cancer care, Abernethy et al [27] showed that an RLHS provided detailed data on the patient’s experience and rapid analysis of feedback to support subsequent care and allowed for continuous monitoring of outcomes to support patient safety, quality of care, and rapport with health practitioners. Although the RLHS has primarily been used in physical health care [26], there has been recent interest in using RLHS methods to treat mental disorders. Several commentaries have been published providing advice on implementing an RLHS in mental health, behavioral health, and substance use [28-30]. Likewise, several recent protocol papers describe ongoing work using an RLHS to improve treatments for epilepsy [31], autism [32], and early intervention for psychosis [33].

An RLHS is an inherently collaborative process that involves coproduction and joint evaluation of new models of care by patients, family members, providers, researchers, and decision makers [29]. Thus, the RLHS may also be beneficial for bridging the gap between research and practice [25]. To facilitate the uptake and use of an RLHS, implementation science methods can be used to guide and document the process [30,31,33].

### Using Implementation Science to Promote New Models of Care

One of the most widely used implementation science frameworks is the RE-AIM (Reach, Effectiveness, Adoption, Implementation, and Maintenance) model [34]. RE-AIM evolved from a need to evaluate health care interventions when conducted under complex situations typically found in a health care setting. Outcomes for each dimension provide researchers and policy makers insight as to the use of an intervention for a certain population and setting. *Reach* is defined by the number, or proportion, of willing participants undergoing the intervention. *Effectiveness* examines the impact of the intervention on individual outcomes. *Adoption* refers to the proportion of organizations that agree to take part in the intervention. *Implementation* is the extent to which a program is adapted, modified, or used as intended within an organization. Finally, *Maintenance* considers the sustainability of the intervention at an individual and organizational level.

Several studies have used the RE-AIM framework to describe and enhance the implementation of PROMs in mental health care. For example, Mascayano et al [35] paired RE-AIM with measurement-based care to evaluate the implementation of early intervention for psychosis program and identified key areas for improvement such as lack of access in rural areas, lack of qualified staff, and an unsustainable funding model. Several teams have also used RE-AIM to facilitate the uptake of PROMs in primary care clinics to screen adolescent and adult patients for potential mental health issues [36,37]. Finally, a protocol paper by Ferrari et al [33] described using RE-AIM to evaluate the impacts of an RLHS designed to improve early intervention for psychosis.

This protocol will use the RE-AIM framework to evaluate the implementation of standardized PROMs delivered using a digital platform and an RLHS as a way to allocate clients to stratified care in an effort to improve mental health outcomes in a specialty clinic for young adults (age 16-24 years). True to the goals of an RLHS, this project entails a collaboration among youth, family members, health care providers, and researchers to co-design a patient-centered system of care that collects data in real time to identify how to continuously improve both processes and outcomes.

## Methods

### Clinical Setting

This study will take place in a clinic that provides specialized mental health services within a large publicly funded provincial health care organization (Alberta Health Services) to emerging adults in Calgary, Alberta, Canada. Emerging adults (age 16-24 years) referred to the clinic are accepted based on whether they require level 3 care (High Intensity Community Based Services) on the Level of Care Utilization System developed by the American Association of Community Psychiatrists [38]. The clinic requires a referral, which may come from a health care provider or self-referral through telephone helplines. The clinic primarily provides psychotherapy (although psychiatric assessment and medication are also available) so clients need to be motivated to engage in treatment and demonstrate sufficient cognitive capacity to benefit from psychotherapy.

### Ethical Considerations

This study was approved by the Conjoint Health Research Ethics Board at the University of Calgary (REB21-0616). Potential participants will be recruited through referral by staff within the clinic. Participants will sign a consent form, which includes study information, procedures, length, potential risks and benefits, confidentiality measures, and information about choosing not to participate or withdrawing from the study with no penalty and no impact on the standard of medical care they receive. Participants receive an electronic gift card as an incentive for participating in this study (CAD $40 [approximately US $30] for completing each assessment). Participant data collected in this study are stored on the Research Electronic Data Capture (REDCap; Vanderbilt University) platform and backed up on the University of Calgary’s secure data storage servers. Data are also stored within the Innowell (Innowell Pty Ltd) platform, which is legally an Alberta Health Services–held repository of patient data that uses encryption to protect data. Data access is limited to clinicians, individual participants, and the research team.

### Key Objectives

The objectives of the study are as follows:

1. Improve treatment selection: Through the use of stratified care, the clinic is seeking to provide the right intensity of service the first time, resulting in better alignment between client needs and services offered.
2. Reduce average wait time (prior to service) and treatment duration (once in service): If the clinic can increase the precision of services by providing higher-intensity services for higher-need clients and lower-intensity services for lower-need clients, the increased efficiency has the potential to reduce wait times and treatment duration.
3. Increase the value of services: The clinic also seeks to understand the value of services provided to increase cost-effectiveness. By tracking client outcomes associated with each treatment, the clinic can identify which services provide the greatest return on investment.
4. Understand barriers and facilitators to implementing standardized PROMs delivered using a digital platform and to stratified care: Knowledge gained about the barriers and facilitators will rapidly be applied to improve organizational, clinical, and youth-related factors to improve care in the clinic.

### Study Design and Phases

#### Overview

**Figure 1:**
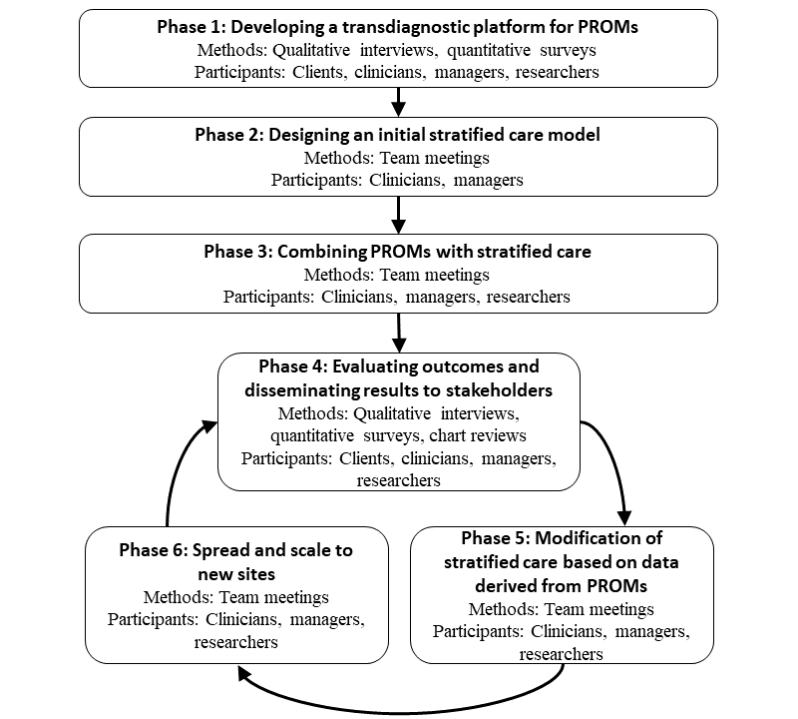
Phases in the development and evaluation of a rapid learning health system using PROMs and stratified care. PROM: patient-reported outcome measure.

The study phases and their relation to an RLHS model are outlined in Figure 1.

#### Phase 1: Developing a Transdiagnostic Platform for PROMs

We set out to select a platform of PROMs, delivered digitally, that would be useful for clinicians in categorizing the severity of mental illness and would be brief and respond to patient-oriented concerns. The goal was to identify a platform that would allow for the simple sharing of information among the relevant stakeholders (clients, clinicians, and researchers) while being compliant with local privacy laws for the storage and distribution of health information. Perhaps most critically, the platform needed to have high acceptability for youth so that they would be willing to complete the instruments.

**Figure 2:**
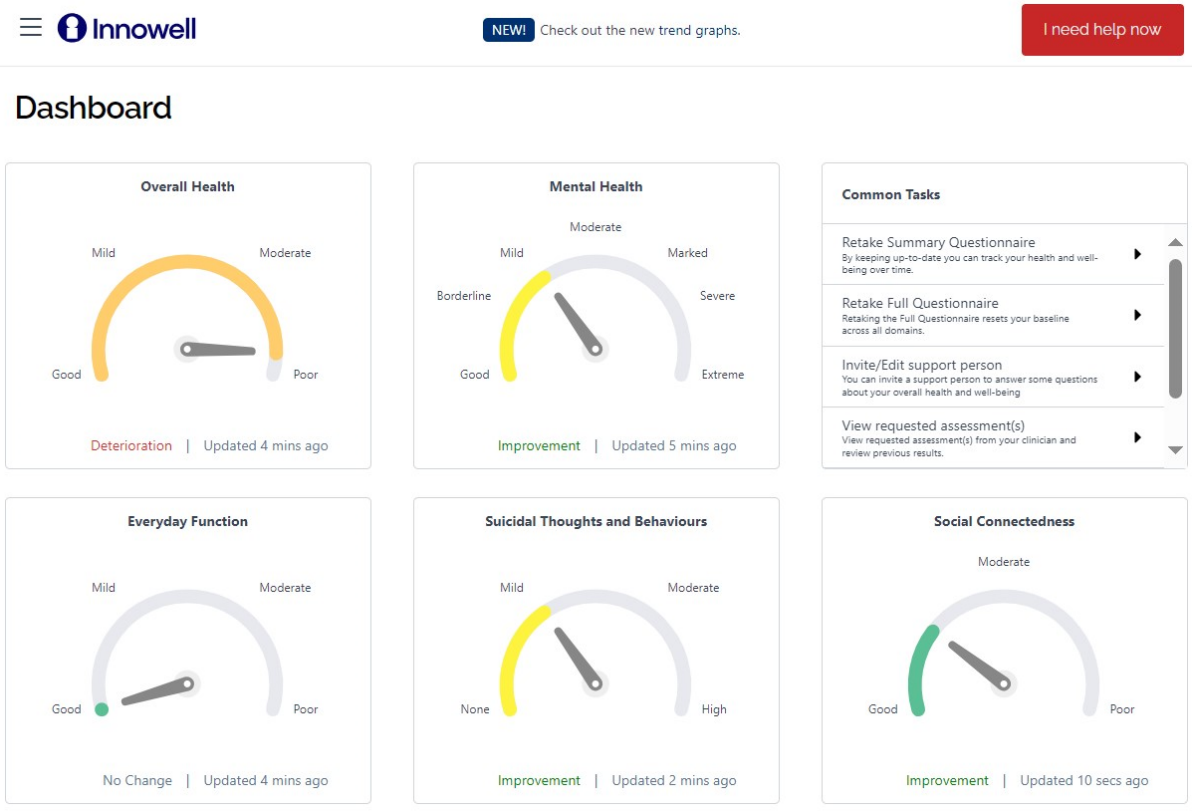
Dashboard for Innowell software used in patient-reported outcome measures among emerging adults to assess disease severity.

We decided to implement Innowell [39], which uses freely available, validated instruments that are part of a curated set of instruments that are embedded in a proprietary web-based platform that was primarily developed by Australian academics with support from the Australian government [39-41]. Raw scores on instruments are converted into categories with clinical interpretations (Figure 2). For example, responses to the Overall Anxiety Severity and Impairment Scale [42] are automatically categorized as “minimal,” “mild,” “moderate,” or “high.” The Innowell platform also allows clients to access resources to work on a specific domain (eg, depressed mood), either by referring them to external resources (typically websites, mobile apps, or phone lines) or allowing them to flag to their provider that they want to address a specific domain.

Clients will complete the Innowell instruments at a minimum of baseline, 6 months, and 12 months (Table 1). If a client is discharged or transferred before 12 months, we will ask them to complete one final assessment at discharge or transfer. Clients can also repeat 1 or more Innowell instruments at any time to gauge treatment progress. All fixed time points except baseline also include a custom instrument to provide feedback on the Innowell system, which includes measures of satisfaction, clinical use, and ease of use. To minimize attrition between time points, we will include the following in the protocol: (1) research coordinator following up with patients to complete measures and sending up to 3 email or text reminders, (2) sharing results with patients including visual graphs so they can see their progress, and (3) providing a small honorarium (in the form of a gift card) for completing measures.

Several additional measures will be collected using the REDCap platform since Innowell does not allow for instrument customization. After each therapy session, clients will be sent an SMS text message (via REDCap) asking them to complete 2 brief 4-item instruments: the Outcome Rating Scale, which measures overall well-being, and the Session Rating Scale, which measures satisfaction with a therapy session [43,44]. At baseline, 6 months, and 12 months, clinicians will be sent an email asking them to provide feedback on the use of PROMs in the Innowell platform and stratified care using the measures listed in Table 2.

**Table 1:** Standardized measures collected from clients. All instruments are administered at baseline, 6 months, and 12 months using the Innowell platform, except for the Outcome Rating Scale and Session Rating Scale, which are collected after each treatment session using the Research Electronic Data Capture (REDCap) platform.

Instruments	Domains
Kessler Psychological Distress Scale	Psychological distress
Overall Anxiety Severity and Impairment Scale	Anxiety
Quick Inventory of Depressive Symptomology	Depressed mood
Prodromal Questionnaire	Psychosis-like experiences
Altman Self-Rating Mania Scale	Mania-like experiences
Primary Care PTSD^a^ Screen for DSM-5^b^	Posttraumatic stress
Eating Disorder Examination	Eating behaviors and body image
Suicidal Ideation Attributes Scale; Columbia Suicide Severity Rating Scale	Suicidal thoughts and behaviors
Brief Non-Suicidal Self-Injury Assessment Tool	Self-harm
Alcohol, Smoking and Substance Involvement Screening Test; AUDIT^c^ Alcohol Consumption Questions	Alcohol, tobacco, and cannabis use
OECD^d^ Youth not in Education or Employment; WHO^e^ Disability Assessment Schedule; Work and Social Adjustment Scale	Social and occupational function
Schuster’s Social Support Scale	Social connectedness
Pittsburgh Sleep Quality Index; Munich Chronotype Questionnaire	Sleep-wake cycle
Child and Youth Resilience Measure—Revised	Resilience
Multigroup Ethnic Identity Measure	Cultural connectedness
Spiritual Health and Life-Orientation Measure	Spirituality
Inventory of Complicated Grief	Grief and loss
Height, weight, and waist circumference; International Physical Activity Questionnaire	Physical health
Outcome Rating Scale	Overall well-being
Session Rating Scale	Treatment session satisfaction

^a^PTSD: posttraumatic stress disorder.

^b^DSM-5: *Diagnostic and Statistical Manual of Mental Disorders, Fifth Edition*.

^c^AUDIT: Alcohol Use Disorders Identification Test.

^d^OECD: Organization for Economic Co-operation and Development.

^e^WHO: World Health Organization.

**Table 2:** Standardized measures collected from clinicians to assess feasibility and satisfaction with the use of patient-reported outcome measures. All instruments are administered at baseline, 6 months, and 12 months using the Research Electronic Data Capture (REDCap) platform.

Instruments	Domains
Attitudes Toward Standardized Assessment Scale	Opinion on psychometric instruments
Evidence-Based Practice Attitudes Scale	Opinion on using evidence-based interventions
Monitoring and Feedback Attitudes Scale	Opinion on routine progress monitoring
Brief Individual Readiness for Change Scale	Capacity to implement change in clinical practice
Implementation Leadership Scale	Capacity to implement evidence-based practice
Factors Associated with Referrals and Holding	Stratified care decision-making

#### Phase 2: Designing an Initial Stratified Care Model

**Figure 3:**
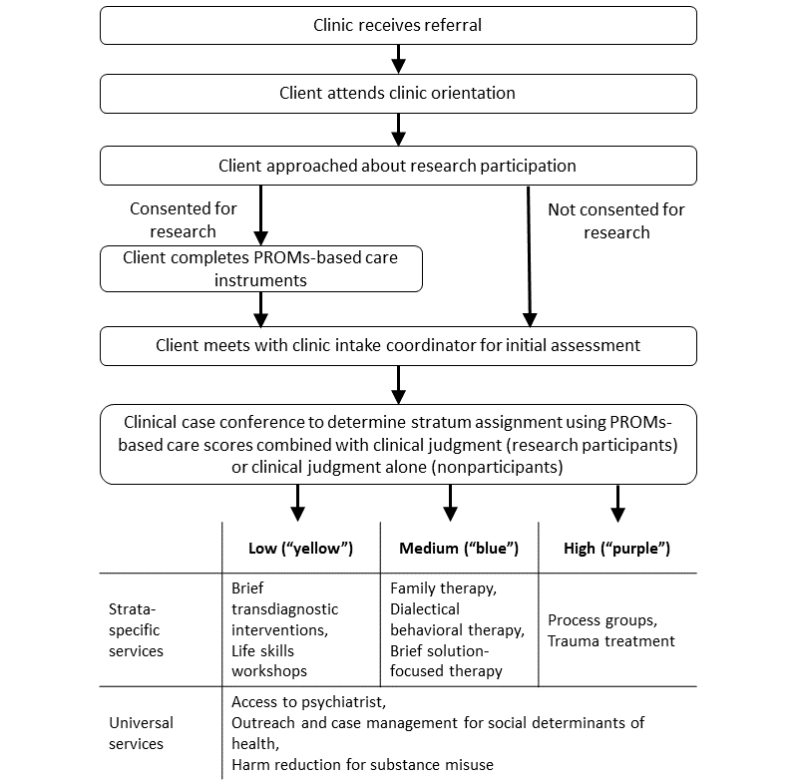
Clinical and research flow diagram showing progression from emerging adult patient intake into clinical strata using PROMs. PROM: patient-reported outcome measure.

Prior to the implementation of an RLHS, standard care at the study clinic was to have clients work with their assigned clinician to select appropriate treatments from among the available options at the clinic. Coincident with the implementation of an RLHS, the clinic made an operational decision to move to a stratified care model, whereby new clients would be appropriately matched to different intensities of care. The optimal timing for PROMs was identified within the clinical workflow: specifically, after a client had received their orientation to the clinic, but before they were assigned to a primary clinician. Managers and clinicians at the study site engaged in multiple rounds of discussion, feedback, and revisions to organize both existing and new services into different strata of care. The initial model includes 3 strata of care (low, medium, and high, which are known in the clinic as “yellow,” “blue,” and “purple,” respectively). Some services are only available to clients in each stratum, while other services are available to all clients regardless of stratum (Figure 3).

#### Phase 3: Combining Implementation of PROMs With Stratified Care

After selecting and implementing a digital platform for the delivery of PROMs and a stratified care model, the next phase was to identify how to use the platform to guide stratification within the study clinic. This involves engaging in rapid learning cycles as the Innowell system was created with a population-wide focus, but the study site serves a much narrower population of emerging adults with moderate to severe mental health concerns. Further, given the clinic-specific stratified care model implemented, further learning cycles were required to determine how to use the results from the selected platform to assign a stratum of care. To accomplish this, at the study site, new clients who consent are onboarded to the Innowell platform and asked to complete all 20 domains available on the platform. In the last 9 months, we introduced the Innowell platform and successfully onboarded 56 clients. Uptake has increased during this time, and at the time of writing, we were onboarding an average of 9 emerging adults per month. Managers and clinicians participate in weekly case conferences where individual clients’ results, referral information, and case history are presented and discussed. Consensus-based decision-making is used to determine the initial client stratum. A researcher will be present during each case conference to capture notes regarding the decision-making process including the key findings used to determine stratum and level of agreement among team members.

As per the stratified model of care, clients will meet with their clinician every 3 months to review treatment progress including current results from readministering all 20 domains. Treatment reviews will be used to determine if clients should be moved to a different stratum of care, transferred to another program, or discharged from the health system. After each clinical stratification treatment review, clients will complete a custom form to provide their feedback on the treatment review and clinical stratification process. We will also conduct a detailed chart review to understand the impact of implementing standardized PROMs and stratified care on the services clients use at the study clinic as well as their use of wider health system resources. This chart review will also include clients who did not consent to research if a waiver of consent can be obtained.

#### Phase 4: Evaluating Outcomes and Disseminating Results to Stakeholders

An exploratory mixed methods approach will be used to evaluate implementation outcomes. Initially, feedback from qualitative interviews will be prioritized since qualitative interviews yield rich data and allow flexibility to probe deeply on specific issues. The first 20 clients to use the digital platform to inform their stratification of care will be approached for qualitative interviews. We will ask clients about their satisfaction with the PROMs and stratified care systems, what factors they felt influenced the stratification decision, the extent to which they agreed with the stratification decision, as well as probe for barriers and facilitators to the use of PROMs and stratified care.

Once 100 clients have been enrolled in the study for at least 3 months (meaning they have received their initial clinical stratification and at least one treatment review), we will begin to use quantitative data to evaluate outcomes of the stratified care process. Based on current rates of onboarding described earlier, we estimate that we will reach this target sample size in approximately 1 year. We will examine the stratum a client is assigned to and map the trajectory of symptoms and satisfaction with clinical services (see instruments in Table 3). Statistical regression models will be developed to describe symptom change over time and determine whether symptom trajectory (eg, slope of regression model) is modified by stratum of care when controlling for other variables (eg, demographics and social determinants of health).

Clients and their clinicians will have access to the data provided by the client to inform future treatment decisions. The research team will present their interim analysis of the results to the clinical team at team meetings at least once every 3 months. One year after recruitment begins, the research team will conduct focus groups and interviews with clinicians and managers at the study site to understand their satisfaction with PROMs and stratified care, barriers and facilitators to the use of PROMs and stratified care, and the factors that influence their decisions about clinical stratification.

**Table 3:** Data collection using RE-AIM (Reach, Effectiveness, Adoption, Implementation, and Maintenance) framework.

RE-AIM category and data source		Items
**Reach (individual)**		
	Clients	The proportion of clients who participate in a research study, complete quantitative surveys, take part in qualitative interviews, and use extra support resources in Innowell platform
	Clinicians	The proportion of clinicians who complete training on using PROMs^a^, participate in a research study, complete quantitative surveys, and take part in qualitative interviews
	Managers	The proportion of managers who complete training on using PROMs, participate in a research study, and take part in qualitative interviews
**Effectiveness (individual)**		
	Admin data (health region)	The proportion of clients who (for psychiatric reasons) visit urgent care center, who visit emergency department, and who are hospitalized
	Admin data (clinic)	Change over time in the proportion of clients assigned to each stratum of care, the proportion of clients who drop out of treatment, length of waitlist, time spent in the program, cost of services used, number of sessions used, type of services accessed, and number and timing of strata changes
	Clients	Change over time in answers to quantitative surveys, reasons for dropping out of research or treatment, confidence managing mental health services, satisfaction with mental health services and care coordination, satisfaction with the use of PROMs and stratified care, individual barriers and facilitators to the use of PROMs and stratified care, and agreement with clinician and data derived from PROMs regarding stratified of care assignment
	Clinicians	Change over time in answers to quantitative surveys, satisfaction with the use of PROMs and stratified care, individual barriers and facilitators to the use of PROMs and stratified care, and agreement with client and data derived from PROMs regarding stratum of care assignment
	Managers	Change over time in satisfaction with the use of PROMs and stratified care and individual barriers and facilitators to the use of PROMs and stratified care
**Adoption (organizational)**		
	Clinics, clients, clinicians, managers	The proportion of clinics that adopt PROMs and stratified care and stakeholders within clinics (clients, clinicians, and managers) who take part in the implementation of PROMs and stratified care
**Implementation (organizational)**		
	Clinics, clients, clinicians, managers	Organizational barriers and facilitators to the implementation of PROMs and stratified care and changes to the use of PROMs and stratified care relative to the intended use
**Maintenance (individual and organizational)**		
	Clinicians, managers, executive leaders	Commitment to continuing using PROMs and stratified care beyond the study period and spread and scale of PROMs and stratified care

^a^PROM: patient-reported outcome measure.

#### Phase 5: Modification of Stratified Care Based on Data Derived From PROMs

Initially, the clinical team will make rapid changes to both the services offered within each stratum of care and the way dashboards displaying data from PROMs are interpreted and used to match clients into a stratum of care. This pilot phase will be used to ensure managers and clinicians can use early learnings to make effective and efficient changes. Once the management and clinical team report that early learnings have plateaued, planned for 3 months into implementation, the team will use the researcher’s summary analysis of case notes from each case conference to create a decision tree to assign patients to appropriate clinical interventions using the stratified care model developed by the clinical team. As an added step before acting upon the decision tree, every 3 months the research and clinical team will meet to examine the data that have been generated and try to understand how they can adapt the decision tree to increase the precision of using data derived from PROMs to assign a client to a stratum of care. The clinical services available in each stratum will also be re-evaluated.

#### Phase 6: Spread and Scale to New Sites

Initially, this research will be conducted at a single clinic to maximize feasibility and allow for careful evaluation of the use of the stratified care model and its integration with the digital PROM platform.

### Data Analysis

We will use the RE-AIM framework to design and organize the data collection system (Table 3). The *Reach* dimension captures individual outcomes and includes the proportion of stakeholders who take part in the research, complete quantitative and qualitative assessments, and use the additional care options, apps, and e-tools on the Innowell platform. The *Effectiveness* domain also examines individual outcomes and measures changes over time in key outcomes related to mental health (among youth clients), satisfaction with PROMs and stratified care (among clients and clinicians), as well as the use of health care resources (from administrative data). *Adoption* and *Implementation* measure organizational outcomes and so will be more important in later phases of the research when examining how many clinics adopt PROMs and stratified care as well as what are the barriers and facilitators to adoption. *Maintenance* has the longest timescale among RE-AIM dimensions, and it will be used to measure the sustainability of implementation of PROMs and stratified care over time at an organizational and individual level.

This study will begin at the same time as the clinic is implementing stratified care for all clients. For clients who consent to research, PROM results will be used to help inform stratified care, whereas nonresearch participants will be stratified based on clinical judgment alone. The impact of stratified care and implementing PROMs will be inferred from longitudinal changes in client and clinic data, although we cannot control for the impact of other variables changing over time. We will seek a waiver of consent to compare research participants to nonresearch participants, which will provide more direct insights into the impact of using PROMs to inform a stratified care model of mental health service delivery. Analysis will be guided by the three key objectives.

1. Improving treatment selection: We will examine changes over time in symptoms for clients at the study clinic. To the extent that implementation of PROMs is leading to continuous improvements in treatment selection as part of an RLHS, we would expect that participants enrolled at the beginning of the study will show slower improvements in their mental health relative to clients enrolled later in the study (as measured by instruments in Table 1). Additionally, if a waiver of consent can be obtained to compare research participants to nonresearch participants, we expect that research participants will show greater improvements in mental health than nonresearch participants when controlling for clinic intake date.
2. Reducing average wait time (prior to service) and treatment duration (once in service): We will examine clinic administrative data to understand whether the implementation of PROMs and stratified care leads to reductions in wait time and treatment duration. If we can obtain a waiver of consent, we will examine whether research participants (completing PROMs using the Innowell platform) spend less time in treatment than those who opt out of research (and do not complete PROMs using the Innowell platform).
3. Increasing the value of services: Clinic administrative data will be used to understand the cost of providing care to each client. When combined with data derived from PROMs, this will provide information about the degree of symptom improvement associated with each type of service. If we can obtain a waiver of consent, we will be able to determine whether the average cost of providing care to a client changes when informed by PROMs. The clinic also seeks to understand the value of services provided to increase cost-effectiveness. By tracking client outcomes associated with each treatment, we can identify which services provide the greatest return on investment.

## Results

This project is funded by the Alberta Children’s Hospital Foundation as one part of a program grant (the “Framework for Research in Emerging Adults”) that is funded from 2021 to 2026. Ethics approval for this study was received in February 2023. Presently, we have developed a system of PROMs and organized clinical services into strata of care. We will soon begin using PROMs to assign clients to a stratum of care and using feedback from youth and clinicians to understand how to improve experiences and outcomes.

## Discussion

### Principal Findings

This protocol paper describes how we will evaluate the implementation of an RLHS to improve service delivery within a stratified model of care. The study clinic is looking for ways to target the right service to the right client at the right time, in line with current movements toward a precision mental health care system [20]. However, standard care at the study clinic has historically been similar to mental health care in most areas of the world, in that, treatment progress is not systematically tracked using objective measures for most clients [19]. Thus, even if the clinic implemented stratified care as a means to tailor services to client needs, the clinic would have no way of knowing if the stratified care model resulted in improved outcomes. Therefore, the clinic needed to develop a platform for PROMs to monitor outcomes to understand the impact of their new stratified care model. Using PROMs to inform stratified care is challenging since there are no existing guidelines to help clinicians understand how scores on different standardized measures translate into treatment recommendations. Therefore, the clinic will need to use an RLHS framework to guide the use of PROMs in informing stratified care decision-making. Initially, stratification using data derived from PROMs (in combination with clinical judgment) may not be superior to stratification using clinical judgment alone. However, as data are collected on how stratification and treatment selection impact the trajectory of a client’s symptoms, the goal is to iteratively improve the ability to stratify clients and select the best treatment plan.

This project has broad implications for mental health care systems as part of a movement toward value-based health care, which is grounded in the notion that compensation for health care services should be based on the amount of benefit to patients rather than the expense incurred by providers and the system [45]. By tracking the relationship between health services and health outcomes, organizations that pay for health care (eg, governments and insurance companies) can obtain an enhanced understanding of which services provide the greatest value for patients at the lowest financial cost [45]. Implementing value-based health care requires a digital platform, patient-centered outcome measurements, tools to support clinical decision-making, and the means to allow for continuous improvement based on data fed into the digital platform [45,46]. An RLHS, such as the one we are implementing and evaluating, leverages these same key components [47] and therefore represents a potentially important first step toward achieving the goal of a value-based health care system [48].

This research will provide practical guidance to help other research and clinical teams collaborate to implement an RLHS as part of the transition toward value-based health care. The engagement of multiple stakeholders early in the design process allowed us to identify the key outcomes that are fundamental toward creating a value-based health care system. Testing out multiple strategies should allow us to identify a system that patients and providers would be willing to use consistently enough to understand which services are having the biggest impact on patient outcomes. Once additional data are generated, we will be able to report on strategies that clinicians use to identify the treatment that is predicted to provide the most value to patients. However, this research protocol focuses on the systematic evaluation of individual outcomes, which is only one component of a value-based mental health care system. At a population level, value-based mental health care also requires additional system-level quality measures including evaluation of structure (eg, number and availability of mental health specialists) and process (eg, number of sessions of psychotherapy) [48].

### Potential Challenges and Limitations

A major uncertainty is the extent to which clinicians will be willing to embrace the use of standardized PROMs to inform decisions about stratified care. Most mental health providers do not use standardized measures and instead rely on their own clinical judgment to determine the best course of care [19]. It will take some time before enough data are collected to allow for past client outcome data to meaningfully inform decisions about future client stratification. The status quo exerts a powerful effect on organizations, and it is common for new mental health initiatives to be abandoned after a few years, especially if there are problems with staff turnover or funding [49,50]. To mitigate these issues, our team spent 3 years building collaborative relationships among researchers, clinic management, and staff through working together on smaller projects, such as a feasibility study of brief transdiagnostic psychotherapy [12]. We are also embedding qualitative and quantitative measures of clinician satisfaction and decision-making throughout the process to identify how we can support clinicians to implement standardized PROMs and stratified care. Another limitation of this protocol is that the decision tree will have limited generalizability to clinics with different patient populations and referral criteria. In the future, we plan to include additional sites to evaluate the approach in other clinical settings serving emerging adults with mental health concerns. Finally, there is the potential bias toward those who contribute the most data [51]. Specifically, emerging adults who consent to completing PROMs and participating in research may not fully represent all patients referred to the clinic. We will evaluate this potential source of bias by analyzing differences between clients who consent to research and those who do based on demographic and clinical variables (eg, diagnoses) available in administrative data.

### Conclusions

**Figure 4:**
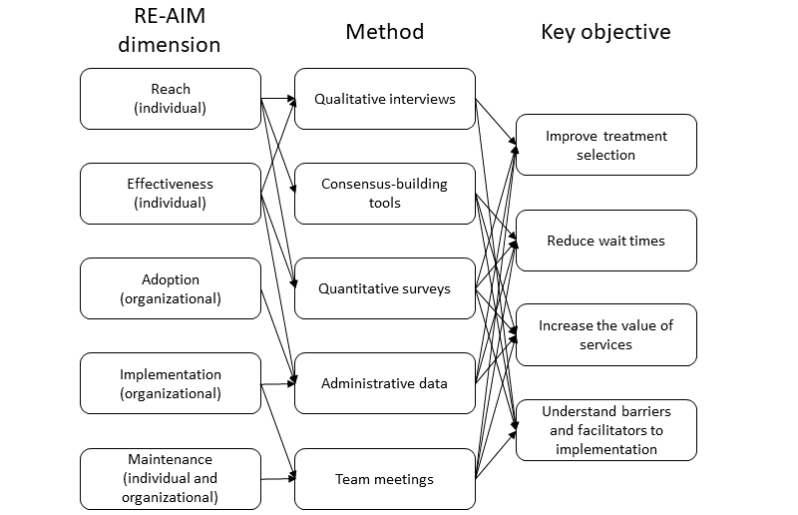
Relationship among RE-AIM framework, research methods, and key objectives. RE-AIM: Reach, Effectiveness, Adoption, Implementation, and Maintenance.

This project aims to implement and evaluate an RLHS and enable a data-driven, stratified care approach to improving emerging adult mental health services. The RE-AIM framework is being used to organize and evaluate the implementation according to the key objectives (Figure 4). The goal of this work is to move away from a “one size fits all” approach to youth mental health services and toward one that customizes the modality and intensity of treatment based on client symptoms and preferences. Now that the initial system has been designed, the immediate next steps are to collect and analyze the outcome data to make iterative improvements in stratification. This study will provide valuable insights for clinicians and researchers who are seeking to use mental health data to improve the allocation and delivery of health care resources in real-world settings.

## Abbreviations

PROM: patient-reported outcome measure
RE-AIM: Reach, Effectiveness, Adoption, Implementation, and Maintenance
REDCap: Research Electronic Data Capture
RLHS: rapid learning health system

## Data Availability

The data sets generated during this study are not publicly available because they contain potentially identifiable health care data but are available from the corresponding author on reasonable request.
